# Targeting Aging‐Related Neuroinflammation and Autophagy as Interventions for Late Onset Alzheimer’s Disease

**DOI:** 10.1002/alz.091053

**Published:** 2025-01-03

**Authors:** Diogo Francisco Silva Santos, Katy A Haynes, Sean‐Paul Gerard Williams, Jenny M Willis, Umesh Nepali, Aman Reddy, Nicholas Heaton, Jason T Hart, Leila Letica, Lauren R Dubberley, Sang‐Ho Choi, David J Schaeffer, Afonso C Silva, Lais da Silva, Paul R Territo, Sara K Quinney, Yuan Liu, Bill Chen, Toren Finkel, Diogo J Sukoff Santos

**Affiliations:** ^1^ University of Pittsburgh, Pittsburgh, PA USA; ^2^ 100 Technology Drive, 100 Technology Drive, PA USA; ^3^ University of Pittsburgh School of Medicine, Pittsburgh, PA USA; ^4^ Indiana University, Indianapolis, IN USA

## Abstract

**Background:**

The bi‐directional autophagy and inflammation network becomes progressively dysregulated with age, with systemic inflammation as a biomarker of this dysregulation including in Alzheimer’s Disease (AD). We hypothesize that interventions which target the shared feature of systemic inflammation in the biology of aging and AD, via regulation of the autophagy‐inflammation network, will prevent the conversion to disease pathogenesis in AD as well as improve healthspan and longevity in aging populations. While previous studies report benefits of mTOR inhibition including rapamycin in transgenic mouse models of familial AD, the present studies aim to evaluate this pathway in a model of sporadic, late onset AD (LOAD) and test the contribution of AMP‐activated protein kinase (AMPK) as a critical regulator of the mTOR pathway.

**Method:**

A novel brain penetrable AMPK activator compound (BC1618) and the prototypical mTOR inhibitor rapamycin were administered chronically to male and female mice with genetic and environmental risk for LOAD. LOAD mice (APOE4/TREM2R47H/hAPP; JAX #030670) were conditioned on High Fat Diet (HFD) from 2 months of age which induced sustained inflammation. Drug exposure levels were measured by HPLC‐MS/MS for PK analysis. Subjects were evaluated through a comprehensive battery of tests longitudinally at 6‐month intervals throughout lifespan including motor, sensorimotor, cognitive behavior, frailty, blood‐based biomarkers, and PET/MR.

**Result:**

Treatment with rapamycin and BC1618 resulted in the expected appreciable exposure levels in brain and plasma. Interim analysis of LOAD+HFD mice at 22 months of age (10+ months treatment) revealed reduced survival and healthspan relative to non‐LOAD controls with no benefit of RAPA or BC1618 treatment. Ongoing studies evaluating translationally relevant PET/MR, biomarkers, pathology, and behavior throughout lifespan to evaluate effects on healthspan and lifespan are in progress.

**Conclusion:**

LOAD+HFD mice model genetic x aging x environmental risk of sporadic late‐onset AD and provide a more translationally relevant model to study potential benefits of interventions that target the mTOR pathway. These studies are poised to inform influence of AMPK on autophagy and inflammation to attenuate disease progression in LOAD; which may be better predictive of effects on sporadic AD than previous studies in young transgenic early‐onset models of brain amyloid overexpression.

